# Increased Formation of Follicular Antrum in Aquaporin-8-Deficient Mice Is Due to Defective Proliferation and Migration, and Not Steroidogenesis of Granulosa Cells

**DOI:** 10.3389/fphys.2018.01193

**Published:** 2018-08-23

**Authors:** Dejiang Wang, Xiangjun Di, Jie Wang, Miao Li, Di Zhang, Yaxin Hou, Jiao Hu, Ge Zhang, He Zhang, Meiyan Sun, Xiangyu Meng, Bo Sun, Chunlai Jiang, Tonghui Ma, Weiheng Su

**Affiliations:** ^1^National Engineering Laboratory for AIDS Vaccine, School of Life Sciences, Jilin University, Changchun, China; ^2^Key Laboratory for Molecular Enzymology and Engineering of the Ministry of Education, School of Life Sciences, Jilin University, Changchun, China; ^3^China-Japan Union Hospital, Jilin University, Changchun, China; ^4^College of Animal Science and Technology, Jilin Agricultural University, Changchun, China; ^5^College of Basic Medical Sciences, Dalian Medical University, Dalian, China; ^6^Department of Laboratory Medicine, Jilin Medical University, Jilin, China

**Keywords:** aquaporin-8, granulosa cells, folliculogenesis, antral follicle, cell proliferation, cell migration, intercellular space

## Abstract

Aquaporin-8 (AQP8) is a water channel protein expressed exclusively in granulosa cells (GCs) in mouse ovary. Our previous studies of AQP8-deficient (AQP8^-/-^) mice demonstrated that AQP8 participates in folliculogenesis, including in the formation of follicles, ovulation, and atresia. However, its physiological function in formation of the antral follicle is still largely unknown. In the present study, we observed significantly increased numbers of antral follicles in AQP8^-/-^ ovaries as well as significantly increased follicular antrum formation in *in vitro* 3D culture of AQP8^-/-^ follicles. Functional detection of AQP8^-/-^ GCs indicated that cell proliferation is impaired with FSH treatment, and wound healing and Transwell migration are also impaired with or without FSH treatment, compared with that in WT. However, the biosynthesis of estradiol and progesterone as well as the mRNA levels of key steroidogenic enzyme genes (*CYP19A1* and *StAR*) in AQP8^-/-^ GCs did not change, even with addition of FSH and/or testosterone. In order to estimate the influence of the impaired proliferation and migration on the density of GC mass, preantral follicles were injected with FITC-dextran, which distributes only in the intercellular space, and analyzed by confocal microscopy. The micrographs showed significantly higher transmission of fluorescence in AQP8^-/-^ follicles, suggesting increased intercellular space of GCs. Based on this evidence, we concluded that AQP8 deficiency leads to increased formation of follicular antra *in vivo* and *in vitro*, and the mechanism may be associated with increased intercellular space of GCs, which may be caused by defective proliferation and migration of GCs. This study may offer new insight into the molecular mechanisms of the formation of follicular antrum.

## Introduction

The formation and expansion of the fluid-filled antrum, as well as the development of the structural foundation of follicular differentiation and ovulation, are important processes during ovarian folliculogenesis. Antrum formation begins with the development of granulosa cells (GCs) in preantral follicles. A few cell–cell contacts between multiple layers of GCs form a small fluid cavity, the first foci of fluid accumulation, and as they expand and coalesce, a larger, centrally located antrum develops (Rodgers and Irving-Rodgers, 2010). However, the mechanisms underlying antrum formation are still largely unknown.

As the antrum begins to appear, specific growth-supporting factors begin to express, including IGF-1, ERa/b subtypes, FSH receptor, and cyclin D2 (Richards, 2001). These expressed proteins lead to expansion of the antrum. After formation, the antrum is filled with follicular fluid, which is secreted by GCs and diffused from the thecal capillaries. Follicular fluid consists of water, low-molecular-weight components, proteoglycan, and enzymes and contains a variety of soluble regulatory factors, such as follicle-stimulating hormone (FSH), luteinizing hormone (LH), estradiol, progesterone, insulin-like growth factor-1, tumor necrosis factor α, interleukin-6 (IL-6), and stem cell factor, which are essential for oocyte maturation and fertilization, GC proliferation, differentiation, luteinization, and follicle ovulation (Zachut et al., 2016).

Inappropriate development of the follicular antrum may result in serious ovarian pathologies such as ovarian cyst, polycystic ovary syndrome (PCOS), and ovarian tumor (Biason-Lauber and Chaboissier, 2015). These diseases are the leading causes of female subfertility and the most frequent endocrine problems in women of reproductive age (Hua et al., 2015). Therefore, it is critical to understand the mechanisms underlying antrum formation for both basic folliculogenesis theory and clinical ovarian pathogenesis.

The aquaporins (AQPs) are a family of water channel proteins that are extensively expressed throughout animals, plants, and lower organisms (Nagaraju et al., 2016). There are 13 mammalian AQPs, whose primary function is to facilitate rapid, passive movement of water molecules. During ovarian folliculogenesis, there is rapid follicular growth with an estimated 19 doublings of follicle surface area (McConnell et al., 2002), and most of that increase is attributed to antral fluid. Previous studies have revealed diverse, important roles of AQPs in ovary and folliculogenesis. Thoroddsen et al. (2011) reported that AQP1–4 are differentially expressed in human granulosa and theca cells of the preovulatory follicle during ovulation and may be involved in follicular rupture and corpus luteum formation. AQP4-deficient mice showed fewer antral follicles and corpora lutea at 8 weeks of age, as well as decreased rates of pregnancy and litter sizes (Sun et al., 2009). It has been demonstrated recently that AQP2 and AQP5 might be involved in efficient water transport through the ovarian bursa, based on the fact that AQP2 is specifically localized in the outer layer (peritoneal side), while AQP5 is localized in the inner layer (ovarian side) of the ovarian bursa in mice (Zhang et al., 2013). AQP7–9 are expressed in rat GCs and may mediate water movement from outside into antral follicles (McConnell et al., 2002). Qu et al. (2010) reported that the expression of AQP9 in human GCs was inhibited by hyperandrogenism in the follicular fluid of women with PCOS through the phosphatidylinositol 3-kinase pathway.

In addition to the aforementioned studies, our group focused on the roles of AQP8 in folliculogenesis and progressively revealed altered physiological functions in ovary and follicle of an AQP8-deficient (AQP8^-/-^) mouse model. We identified the expression of AQP5, AQP7, AQP8, AQP11, and AQP12 in mouse GCs (Su et al., 2013) and revealed that the membrane water permeability of GCs isolated from AQP8^-/-^ mice decreased significantly compared with that of wild-type (WT) (Su et al., 2010). The apoptosis rate of AQP8^-/-^ GCs was also reduced because of slowed apoptotic volume decrease. The reproductive influence of AQP8 deficiency in female mice includes a significant increase in the ovulatory follicles, released oocytes, and fertility (Li et al., 2016). Furthermore, AQP8 deficiency leads to the increased occurrence of multi-oocyte follicles, which distribute at various follicle stages in ovary (Su et al., 2013). Subsequently, another group provided evidence suggesting that AQP8 is associated with human folliculogenesis. Li et al. (2013) described that a single-nucleotide polymorphism (rs2287798) within the human *AQP8* gene exhibits a significantly higher allele frequency in PCOS cases compared with that in controls, implying a possible important role of AQP8 in the pathogenesis of PCOS. In the present study, we evaluated the characteristics of the follicular antrum in AQP8-deficient mice by ovary sectioning and staining, and observed a phenotype with a significantly high number of follicular antrums. A 3D follicular culture approach was employed to represent and confirm the antrum formation process *in vitro*. Then, cell proliferation, migration, and steroid secretion assays on GCs, and measurement of the intercellular space among GCs, were carried out to elucidate a novel mechanism underlying follicular antrum formation.

## Materials and Methods

### Mice

AQP8^-/-^ mice were generated by targeted gene disruption as described in a previous study (Yang et al., 2005). All experiments were performed on age-matched WT and AQP8^-/-^ mice in a C57BL/6 genetic background. The study group comprised of AQP8^-/-^ mice, and the control group of wild-type mice. AQP8^-/-^ mice used in all experiments are homozygous amphiploid genotype. The mice were maintained in a specific pathogen-free animal facility on a 12-h light/dark cycle. Protocols for mouse experiments were approved by the Committee on Animal Research of Jilin University [SCXK (J) 2009-0004].

### Outcome Measures

We hypothesized that AQP8 deficiency would lead to increased formation of follicular antra and change the function of GCs. Ovary sectioning and staining, 3D follicular culture and cell proliferation, migration were the primary endpoints, and steroid secretion assays on GCs and measurement of the intercellular space among GCs were the primary endpoints.

### Ovary Sectioning and HE Staining

Ten ovaries from five 4-week-old mice for each genotype were serially sectioned at a thickness of 4 μm and processed for hematoxylin and eosin (HE) staining by standard procedures. The number of antral follicles for each ovary was counted and summed up from 10 sections with equal interval. Only healthy antral follicles with an obvious oocyte were recorded, according to features described previously (Cheng et al., 2002). The diameter of a follicle was measured by an average of the longest side and shortest side and was evaluated by two independent investigators.

### 3-Dimensional (3D) Culture of Follicle in an Alginate Matrix

Ovaries from 12∼16-day-old female WT and AQP8^-/-^ mice (*n* = 8 for each genotype) were excised, rinsed three times with phosphate-buffered saline (PBS), and then transferred to Leibovitz-15 (L-15) medium (Sigma-Aldrich, St. Louis, MO, United States) containing 1% fetal bovine serum (FBS). The ovaries were punctured by a sterile needle. Preantral follicles with two-layered or multi-layered GCs were isolated using a hand-pulled micropipette and incubated in α-Minimum Essential Medium (α-MEM) (Sigma-Aldrich) with 1% FBS at 37°C and 5% CO_2_ for 2 h.

Sodium alginate (Sangon, Shanghai, China) was dissolved in deionized water to the concentration of 1%, sterilized by filtration, and then reconstituted in PBS to the concentration of 0.5%. A single preantral follicle was transferred into a 3 μL droplet of alginate solution on a polypropylene mesh (100 μm). The mesh with alginate droplets was then immerged into the encapsulation solution containing 50 mM CaCl_2_ and 150 mM NaCl. The follicles in the alginate droplets were cultured in 96-well microplates with α-MEM containing 10 mIU/mL recombinant human FSH (ProSpec-Tany, Rehovot, Israel), 1 mg/mL fetuin (Sigma-Aldrich), 5 μg/mL insulin (Sigma-Aldrich), 5 μg/mL transferrin (Sigma-Aldrich), 5 ng/mL selenium (Sigma-Aldrich), and 3% bovine serum albumin (Sangon) and incubated at 37°C with 5% CO_2_ for 12 days. Half the medium volume was changed, and the follicles were photographed every day. Follicles were considered dead if the oocyte was no longer surrounded by a GC layer or if the GCs had become dark and fragmented as described previously (West-Farrell et al., 2009). Survival rate and antral follicle formation rate were normalized by the ratio of AQP8^-/-^ follicle to WT follicle. The diameter of a follicle was also measured by an average of the longest side and shortest side and was evaluated by two independent investigators.

### Cell Proliferation Detection

4-week-old female WT and AQP8^-/-^ mice were injected intraperitoneally with 10 IU PMSG (ProSpec-Tany). After 48 h, ovaries were excised, rinsed three times with PBS, and then transferred to McCoy’s 5a medium (Sigma-Aldrich) containing 10% FBS. The GCs coming from antral and preantral follicles were freshly harvested from the follicles of ovaries by needle puncture, treated with 0.5% hyaluronidase for 10 min, centrifuged, and resuspended in McCoy’s 5a medium. Then, the cells were seeded in microplates according to the following manipulations.

Freshly isolated GCs from 4-week-old mice (*n* = 10 for each genotype) were seeded in 48-well microplates at a density of 1.5 × 10^5^ cells/well and incubated in McCoy’s 5a medium for 24, 48, 72, and 96 h to monitor cell proliferation. FSH was used as an optional supplement at a working concentration of 100 mUI/mL. At each reading timepoint, CellTiter-Glo^®^ reagent (Promega, Madison, WI, United States) was added to the wells, and the luminescence signals were measured immediately using PerkinElmer VICTOR^TM^ X2 (Waltham, MA, United States) to determine the cell viability. Six wells were measured for each group, and the assay was repeated three times.

### GCs Migration Assay: Wound Healing and Transwell Cell Migration

Freshly isolated GCs from 4-week-old mice (*n* = 8 for each genotype) were seeded in 12-well microplates at a density of 5 × 10^5^ cells/well and cultured until confluent. The cells were wounded by removing a 300–500-μm strip of cells across the well with a standard 100-μL pipette tip. The wounded monolayers were washed twice to remove non-adherent cells and cultured in McCoy’s 5a medium with 2% FBS. FSH was used as an optional supplement at a working concentration of 100 mIU/mL. Then, time-lapse photography of the wound edges was performed at 0 and 48 h. The cell migration rate was quantified as the average linear speed of the wound edges growth. Three wells were measured for each group, and the assay was repeated three times.

The Transwell assays were performed with a modified Boyden chamber (Corning, NY, United States) containing a gelatin-coated polycarbonate membrane filter (6.5-mm diameter, 8-μm pores). The upper chamber contained freshly isolated GCs at a density of 1.5 × 10^5^ cells/well in McCoy’s 5a medium with 1% FBS, and the lower chamber contained McCoy’s 5a medium with 10% FBS. The GCs were collected from six mice for each genotype. FSH was used as an optional supplement in both the upper and lower chamber at a working concentration of 100 mIU/mL. After a 24-h culture, non-migrated cells were scraped from the upper surface of the membrane with a cotton swab. Migrated cells remaining on the bottom surface were counted in five predetermined fields after staining with crystal violet. Three samples were measured for each group, and the assay was repeated three times.

### Steroid Secretion Analysis

Freshly isolated GCs from 4-week-old mice (*n* = 8 for each genotype) were seeded in 12-well microplates at a density of 5 × 10^5^ cells/well and cultured until confluent and cultured in McCoy’s 5a medium with 10% FBS. The culture medium was replaced by serum-free medium supplemented with 10 μg/mL insulin, 5.5 μg /mL transferrin, and 5 ng/mL selenium when cell density reached 80%. After 12-h culture, serum-free medium supplemented with or without 100 mIU/mL FSH and/or 1 μM testosterone (Sigma-Aldrich) replaced the previous medium. Forty-eight h later, the medium was collected and centrifuged. The estradiol and progesterone concentrations in the supernatant were measured using mouse estrogen and progesterone ELISA kits (Cusabio Biotech Co., Wuhan, China), respectively, according to the instructions (Sun et al., 2014). Meanwhile, cells in each well were divided into two equal portions. One portion was used to extract total RNA, and the other was used to measure the total protein content with a BCA protein quantitation kit (KeyGEN, Nanjing, China). The estradiol and progesterone levels were normalized by the ratios of estradiol/progesterone concentration to total protein content. Four samples were measured for each group, and the assay was repeated twice.

### qRT-PCR Assay

Total RNA samples from GCs were freshly extracted using a total RNA isolation kit (TIANGEN, Beijing, China). qRT-PCR was performed using a one-step PrimeScript^®^ RT-PCR Kit (Takara Bio, Otsu, Japan) and CFX96 real-time PCR detection instrument (Bio-Rad, Hercules, CA, United States), and the PCR products were confirmed by agarose gel electrophoresis. The primer sequences were as follows: *StAR*, forward 5′-CCACCTGCATGGTGCTTCA-3′ and reverse 5′-TTGGCGAACTCTATCTGGGTCTG-3′; *CYP19a1*, forward 5′-TGTGTTGACCCTCATGAGACA-3′ and reverse 5′-CTTGACGGATCGTTCATACTTTC-3′; β-actin, forward 5′-CCACCATGTACCCAGGCATT-3′ and reverse 5′-CGGACTCATCGTACTCCTGC-3′. Relative gene expression levels were presented by the comparative cycle threshold (ΔΔCt) method (Su et al., 2010). Briefly, the expression of the target gene was normalized to the endogenous control (β-actin) by subtracting the Ct value of the target gene from the Ct value of the endogenous control. To compare levels relative to a calibrator (mean ΔCt for the WT group without supplements), ΔCt of other groups was subtracted from the calibrator. Relative expression level is given by 2^-ΔΔCt^.

### Measurement of Intercellular Space of GCs by Confocal Microscopy

Fluorescein isothiocyanate (FITC)-dextran (Sigma-Aldrich) of 40-kDa molecular weight was used as an intercellular space indicator that binds to membrane molecules for several hours once it has leaked into junction spaces. Multilayered secondary follicles (200–250 μm in diameter) were mechanically isolated from the ovaries of 4-week-old female WT and AQP8^-/-^ mice (*n* = 6 for each genotype). The glass injection needle at a diameter of 20 μm used for the FITC-dextran injection was pulled by the micropipette puller (Sutter, Novato, United States). The single-follicle injection manipulation was carried out in a microinjector device (Narishige, Tokyo, Japan). Two microliters FITC-dextran (10 μM) was pipetted into the middle of follicles. Then, several slices of each follicle in the middle portion were quickly imaged using a confocal LSM 710 microscope (Carl Zeiss, Hertfordshire, United Kingdom). The relative estimation of intercellular space of GCs was quantified by mean FITC fluorescent intensity of two slices from each follicle using ImageJ software. Results were expressed as fluorescence intensity per μm^2^. *N* = 20 follicles for each genotype, and the experiments were repeated in duplicate.

### Statistical Analysis

Statistical analysis was performed using GraphPad Prism 5 software for Windows (GraphPad Software, San Diego, CA, United States). Grubb’s test for outliers was performed to determine the inclusion or exclusion of data within groups for all datasets. All values are expressed as mean ± SD. The statistical tests were selected based on the normality of the distribution, sample size, and the similarity in variance between groups. The statistical significance of the normal populations was determined by a two-tailed Student’s *t*-test. Differences with a corresponding *P*-value of either <0.05 or <0.01 were considered statistically significant for all experiments. The experiments were repeated three or four times as indicated in the figure legends.

## Results

### Histological Evaluation of Antral Follicles in Ovaries

**FIGURE 1:**
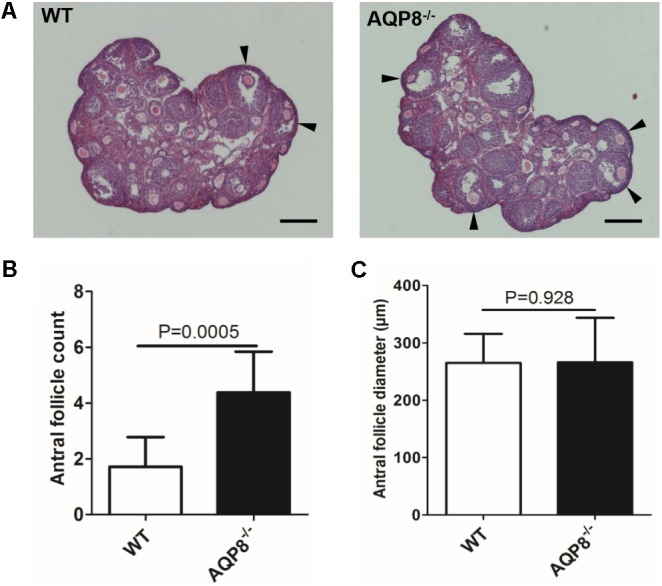
Morphology, quantity, and size of antral follicles in WT and AQP8^-/-^ ovaries. **(A)** Representative images of HE-stained ovary sections. Scale bar, 400 μm. Arrowheads mark healthy antral follicles. **(B)** Quantity of healthy antral follicles per ovary (*n* = 10 ovaries). **(C)** Diameter of healthy antral follicles (*n* = 10 ovaries).

To evaluate the *in vivo* development pattern of follicular antra due to AQP8 deficiency, we first analyzed the number and size of antral follicles in AQP8^-/-^ and WT mice at 4 weeks of age using an ovary serial sectioning and HE staining approach (**Figure 1A**). There was a significant increase in the number of non-atretic antral follicles in the AQP8^-/-^ ovaries when compared with those in WT (4.38 ± 1.46 vs. 1.72 ± 1.60 per ovary, *p* = 0.0005) (**Figure 1B**). However, the size of the non-atretic antral follicles in the AQP8^-/-^ ovaries did not change (266.1 ± 77.7 μm vs. 265.1 ± 50.7 μm, *p* = 0.928) (**Figure 1C**).

### Antral Follicular Growth and Antra Formation in 3D Culture

**FIGURE 2:**
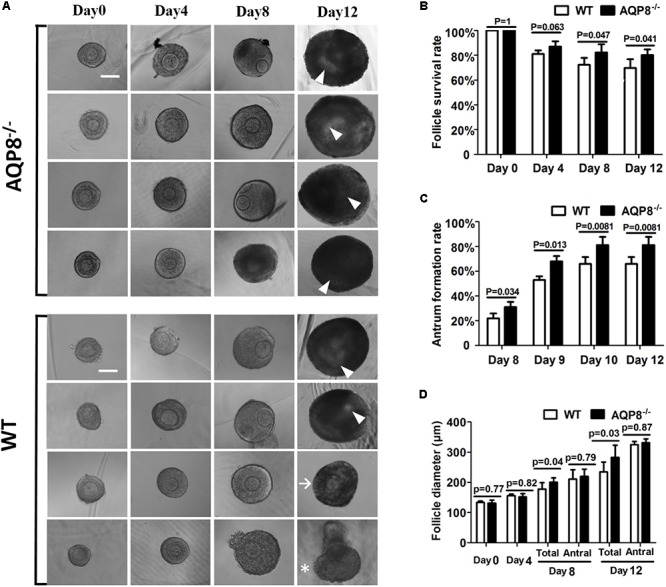
Morphology, survival rate, antrum formation rate, and growth of *in vitro* 3D culture of preantral follicles isolated from WT and AQP8^-/-^ ovaries. **(A)** Representative images of growing follicles at day 0, 4, 8, and 12 of culture in a 3D alginate matrix. Scale bar, 100 μm. Arrowhead marks the antrum; arrow marks surviving follicle without antrum formation; asterisk marks dead follicle. Follicle survival rates **(B)**, antrum formation rates in surviving follicles **(C)**, and follicle diameters **(D)** of 140 AQP8^-/-^ and 126 WT initially cultured follicles are presented at the indicated timepoints. “Total” represents both antral and non-antral live follicles; “antral” represents antral follicles only.

To analyze the *in vitro* antrum formation and growth of antral follicles under AQP8 deficiency, we performed 3D culture of preantral follicles in an alginate matrix (**Figure 2A**). In total, 140 AQP8^-/-^ and 126 WT preantral follicles were isolated and initially cultured in three independent experiments, with the aim of inducing formation of antral follicle. We did not observe significant differences in survival rate during the 4-day culture; However, a significantly higher number of AQP8^-/-^ follicles had survived compared with that of WT on day 8 (follicle survival rate, 0.823 ± 0.0660 vs. 0.7231 ± 0.0556, *p* = 0.047) and day 12 (follicle survival rate, 0.802 ± 0.0450 vs. 0.6980 ± 0.0700, *p* = 0.041) (**Figure 2B**). After 7 days of culture, antra seemed to be observed in some follicles of both genotypes. Among surviving follicles, more antra had formed in AQP8^-/-^ follicles compared with that in WT follicles on day 8 (antrum formation rate, 0.310 ± 0.0420 vs. 0.2200 ± 0.0400, *p* = 0.034), which lasted until the end of culture (antrum formation rate, 0.812 ± 0.0660 vs. 0.6600 ± 0.0556, *p* = 0.0081) (**Figure 2C**). Then, the diameters of the antral follicles were measured. The results indicated no significant difference between follicle size of the two genotypes at day 0, and 4. At day 8 (177.87 ± 21.26 vs. 200.87 ± 13.84 μm, *p* = 0.041) and day 12 (282.54 ± 40.76 vs. 234.88 ± 32.56 μm, *p* = 0.033), the diameters of all the AQP8^-/-^ follicles was significantly higher compared with those of all the WT follicles because of increased survival and antrum formation. However, the diameters of the antral AQP8^-/-^ follicles were approximately equal to that of the antral WT follicles (day 8, 210.87 ± 30.83 vs. 219.87 ± 23.84 μm, *p* = 0.794) and (day 12, 331.66 ± 12.47 vs. 324.75 ± 11.09 μm, *p* = 0.872) (**Figure 2D**). Further, the diameters of oocytes in follicles of both genotypes did not change significantly throughout the 12-day culture (oocyte diameter in AQP8^-/-^ mice was 69.64 ± 1.61 μm at day 1, 73.07 ± 2.44 μm at day 8, 74.87 ± 5.84 μm at day 12; In wild-type mice was 68.40 ± 1.85 μm at day 1, to 72.43 ± 2.35 μm at day 8, 74.00 ± 5.27 μm at day 12).

### Defective Proliferation of AQP8^-/-^ GCs

**FIGURE 3:**
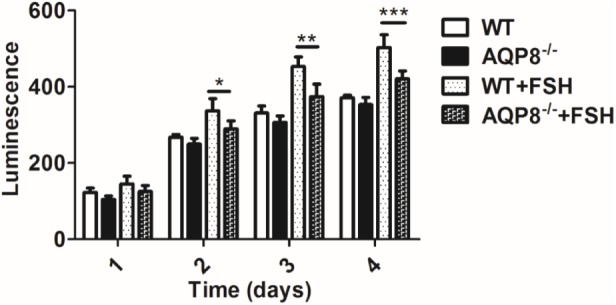
Proliferation measurement of WT and AQP8^-/-^ GCs with or without FSH treatment. The GCs were cultured *in vitro* for 4 days and measured for proliferation each day. *N* = 6 wells for each group. ^∗^*P* < 0.05, ^∗∗^*P* < 0.01, ^∗∗∗^*P* < 0.001.

The proliferation of GCs is the basis of follicle development and is regulated by FSH (Terauchi et al., 2016). Because AQP8 is expressed in GCs, we evaluated the potential impact of AQP8 in proliferation and the cooperation with FSH-induced proliferation of GCs. GCs of AQP8^-/-^ and WT were isolated and cultured with or without FSH for 24, 48, 72, and 96 h, and then the cell viability was measured (**Figure 3**). In groups without FSH treatment, there was no significant difference between AQP8^-/-^ and WT GCs at any timepoint. In groups with added FSH, the proliferation of GCs of both genotypes was promoted. However, AQP8^-/-^ GCs proliferated much slower than WT under the influence of FSH at 48 h (Luminescence, 289.44 ± 21.44 vs. 336.94 ± 31.82, *p* = 0.015), 72 h (Luminescence, 374.15 ± 32.80 vs. 452.73 ± 25.76, *p* = 0.001), and 96 h (Luminescence, 420.96 ± 20.50 vs. 502.52 ± 33.54, *p* = 0.0008).

### Defective Migration of AQP8^-/-^ GCs

**FIGURE 4:**
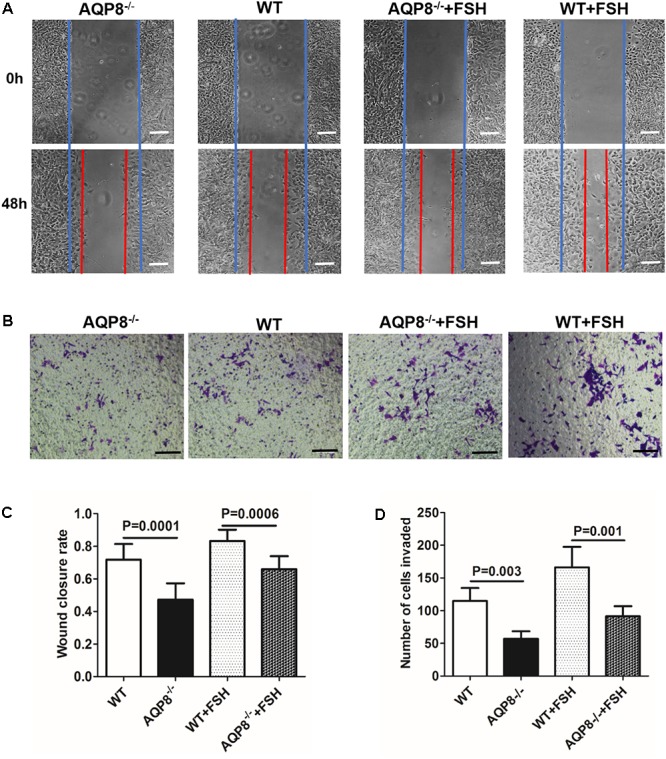
Measurement of wound healing and Transwell migration of WT and AQP8^-/-^ GCs with or without FSH treatment. **(A)** Representative images of wound closure of cultured GCs at 0 and 48 h (blue line, initial wound edge; red line, wound edge after 48 h). Scale bar, 100 μm. **(B)** Representative images of crystal violet-stained GCs in Transwell migration assay after 24 h. Scale bar, 100 μm. **(C)** Wound closure rate in healing experiment (*n* = 3 wells per group). **(D)** Number of invaded GCs in Transwell migration experiment (*n* = 4 wells per group).

The migration of GCs is also essential in follicle development (Buensuceso and Deroo, 2013). AQPs have been proven to facilitate cell migration (Saadoun et al., 2005); therefore, we evaluated the potential impact of AQP8 deficiency in migration and the cooperation with FSH-induced migration of GCs. Wound healing and Transwell migration assays were performed on AQP8^-/-^ and WT GCs with and without FSH treatment. In the wound healing assay, AQP8^-/-^ GCs migrated significantly more slowly than WT without FSH treatment (wound closure rate, 0.47 ± 0.09 vs. 0.72 ± 0.10, *p* = 0.0001) (**Figure 4C**). FSH promoted the migration of GCs of both genotypes, and AQP8^-/-^ GCs migrated significantly slower than the GCs in WT (wound closure rate, 0.66 ± 0.08 vs. 0.83 ± 0.07, *p* = 0.0006). A similar pattern was observed in the Transwell migration assay (**Figure 4D**). Without FSH treatment, the number of AQP8^-/-^ GCs that passed through the chamber decreased significantly compared with that of WT (57 ± 11.42 vs. 114.8 ± 19.77 per well, *p* = 0.003). FSH promoted the passing of the GCs of both genotypes, and AQP8^-/-^ GCs passed significantly slower than the GCs of WT (91.4 ± 15.5 vs. 166.2 ± 31.54 per well, *p* = 0.001) (**Figure 4D**).

### Secretion Levels of Estradiol and Progesterone and Expression Levels of *CYP19A1* and *StAR* mRNA in Cultured AQP8^-/-^ GCs

**FIGURE 5:**
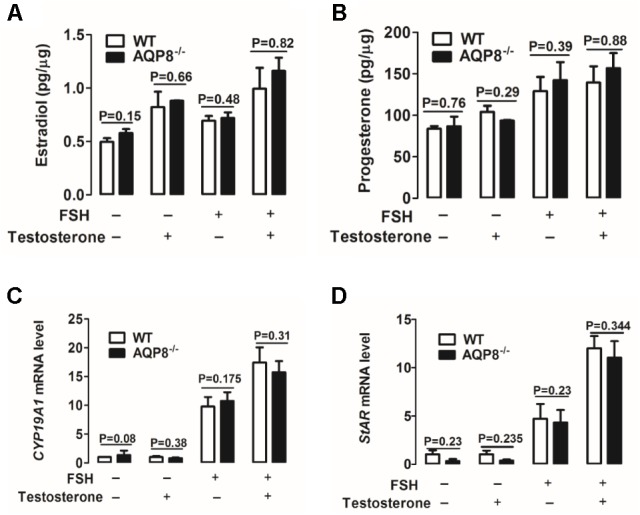
Detection of steroidogenesis and mRNA expression of related genes of WT and AQP8^-/-^ GCs. Biosynthesis of estradiol **(A)** and progesterone **(B)** in cultured WT and AQP8^-/-^ GCs with or without FSH treatment and/or testosterone. *N* = 5 wells per group. Expression levels of CYP19A1 **(C)** and StAR **(D)** mRNA in cultured WT and AQP8^-/-^ GCs with or without FSH treatment and/or testosterone. *N* = 5 wells per group.

Steroidogenesis of GCs is important in the proliferation and differentiation of GCs, maintenance of oocyte growth, and microenvironment homeostasis; therefore, it plays a vital role in folliculogenesis (Atwood and Vadakkadath Meethal, 2016). Estradiol and progesterone are two major steroid hormones synthesized in GCs (Atwood and Vadakkadath Meethal, 2016) and were detected in this study. We examined the effect of AQP8 deficiency on estradiol and progesterone secretion in cultured GCs incubated with and without FSH and/or testosterone, which is a substrate of estradiol biosynthesis (Ishihara et al., 2016). As shown in **Figures 5A,B**, FSH accelerated the production of estradiol and progesterone in GCs of both genotypes. Testosterone rapidly accelerated the production of estradiol only (**Figure 5A**) and slowly promoted the secretion of progesterone (**Figure 5B**). However, none of the experimental groups exhibited a significantly difference in estradiol or progesterone production between AQP8^-/-^ and WT GCs (**Figures 5A,B**).

To further confirm the pattern of steroidogenesis in AQP8^-/-^ GCs, we investigated the expression of enzyme genes participating in estradiol and progesterone biosynthesis. Cytochrome P450 family 19 subfamily A member 1 (CYP19A1) is a key enzyme expressed in GCs and represents the rate-limiting step in the conversion of androgens to estrogens (Kuo et al., 2012; Dou et al., 2016). Steroidogenic acute regulatory protein (StAR) is a key rate-limiting enzyme in progesterone biosynthesis of GCs, because it transports cholesterol from the outer to inner mitochondrial membrane (Yu et al., 2005; Li et al., 2016). The mRNA expression levels of *CYP19A1* and *StAR* in cultured GCs were measured by qRT-PCR. The results indicated that FSH increases the mRNA levels of both genes in GCs of both genotypes (**Figures 5C,D**); testosterone increases the expression of these genes only in the presence of FSH (**Figures 5C,D**). However, no significant difference was found in target mRNA expression between AQP8^-/-^ and WT in any experimental group (**Figures 5C,D**). These results suggested that steroidogenesis is not affected in AQP8^-/-^ GCs.

### Increased Intercellular Space of GCs in AQP8^-/-^ Follicles

**FIGURE 6:**
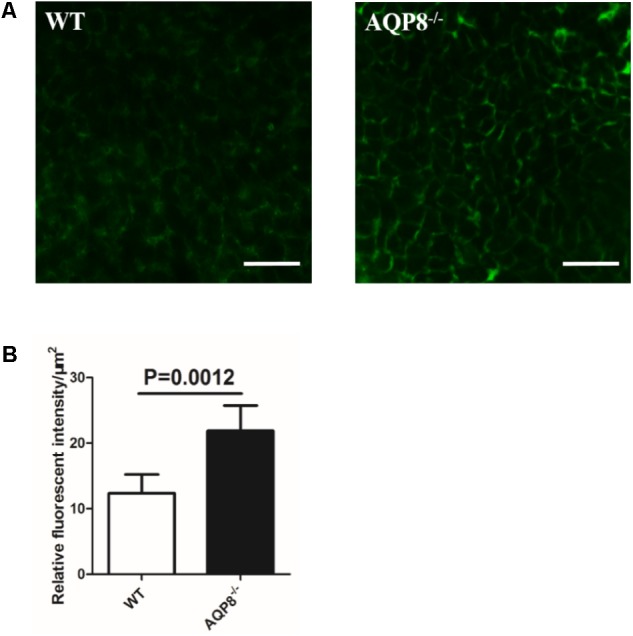
Determination of FITC-dextran fluorescence intensity in intercellular space of WT and AQP8^-/-^ follicles. Preantral follicles were freshly isolated, injected with FITC-dextran, and analyzed by confocal microscopy quickly. **(A)** Representative confocal microscopy images of WT and AQP8^-/-^ follicles, indicating clear cell-cell intercellular space. Scale bar, 25 μm. **(B)** The intercellular space among GCs was evaluated by quantification of FITC fluorescence intensity. Results are expressed as fluorescence intensity per μm^2^. *N* = 20 in two independent experiments.

Given the observations of unaltered size of AQP8^-/-^ follicles and defective proliferation and migration of AQP8^-/-^ GCs, we sought to determine whether the density of GC mass is lower or the intercellular space is wider in AQP8^-/-^ follicles. Preantral follicles were freshly isolated, injected with FITC-dextran of 40-kDa molecular weight, which is an intercellular space stain that binds to membrane molecules for several hours, and then analyzed by confocal microscopy quickly (**Figure 6A**). The FITC fluorescence intensity was quantified to estimate the intercellular space. We found that the relative intensity of FITC fluorescence in AQP8^-/-^ follicles was significantly stronger compared with that of WT (Relative fluorescence intensity, 21.87 ± 3.847 vs. 12.36 ± 2.838, *p* = 0.0012) (**Figure 6B**), suggesting increased intercellular space among GCs in the AQP8^-/-^ follicles.”

## Discussion

In the present study, we demonstrated that AQP8 deficiency causes significantly increased numbers of antral follicles with unaltered follicle size in mice both *in vivo* and *in vitro*. In previous studies, our group has revealed increased follicular ovulation and increased formation of multi-oocyte follicles in AQP8-deficient mice (Su et al., 2010; Su et al., 2013). In mouse ovary, AQP8 has been proven to be exclusively expressed in GCs (Su et al., 2013). These results suggested that the loss of AQP8 function in ovary causes the increased formation of follicular antrum.

In our 3D culture of preantral follicles, AQP8^-/-^ follicles showed a significantly increased survival rate compared with that of WT (**Figure 2B**). This result is consistent with our previous study, in which the apoptosis rate of GCs was decreased by AQP8 deficiency (Su et al., 2010), because the atresia or death of a follicle is primarily dependent on the apoptosis of GCs (Hughes and Gorospe, 1991; Jiang et al., 2003).

Granulosa cells play a key role in successful formation of a mature follicle (Skinner et al., 2008). The growth of a follicle depends on proliferation and migration of GCs, and the maturation of an oocyte depends on the support of factors derived from GCs and stimulation of sex hormones, which are mainly secreted by GCs. We demonstrated that some GC functions are altered because of AQP8 deficiency in this study. The proliferation of AQP8^-/-^ GCs was decreased compared with that in WT, only if FSH was present (**Figure 3**). Some AQPs were reported to participate in cell proliferation by regulating cell volume (Huang et al., 2013; Galan-Cobo et al., 2016; Ni et al., 2016). However, our results provided evidence that AQP8 does not influence GC proliferation directly but is involved in FSH-induced GC proliferation. The underlying mechanisms need to be further explored. The migration of AQP8^-/-^ GCs decreased regardless of the existence of FSH compared with that in WT (**Figure 4**). Similar roles of AQPs in cell migration were also illustrated in previous studies. Saadoun et al. (2005) indicated that AQPs polarized at the leading edge of the prominent membrane ruffles of the migrating cells and facilitated rapid water fluxes, which is beneficial for cell migration. Steroidogenesis is one of the most essential functions of GCs and ovary, but we demonstrated that AQP8 does not play roles in biosynthesis of estradiol and progesterone in GCs. The levels of these hormone secretions and the expression of related biosynthesis enzymes did not change in AQP8^-/-^ GCs (**Figure 5**). Our results have confirmed that AQP4 and APQ8 play completely different roles in the female reproductive system—AQP4 is expressed in the mammalian brain where it regulates the functions of sex hormones, whereas AQP8 is expressed in the GCs in ovaries and is a direct participant in folliculogenesis (Sun et al., 2009). Furthermore, we investigated the intercellular space in preantral follicles and revealed that it is increased in AQP8^-/-^ follicles compared with that in WT (**Figure 6**). This result is important to support our novel finding. However, the detailed mechanics of increased intercellular space in preantral follicles of AQP8^-/-^ mice are not fully understood and need to be covered in future research.

The formation of antrum is a crucial event in the progression of folliculogenesis. During this event, a number of important changes take place in follicles (Rodgers and Irving-Rodgers, 2010). In GC masses of a preantral follicle, multiple small foci appear first, then become fluid-filled spaces, and eventually coalesce to form a single antral cavity (Rodgers and Irving-Rodgers, 2010). However, the force that drives the formation of antrum and the underlying mechanisms are not well understood.

To explain the mechanism of follicular antrum formation, one hypothesis suggests that antrum can be formed by GC death, which provides space for foci development and fluid accumulation (Rolaki et al., 2005; Rodgers and Irving-Rodgers, 2010). However, there is no experimental evidence to prove this hypothesis. In addition, the results in this study did not support this hypothesis. Our previous study demonstrated that AQP8 deficiency decreases the apoptosis of GCs (Su et al., 2010). The underlying mechanism has been revealed by Jablonski et al. (2004). AQP-mediated water loss is important for apoptotic volume decrease of GCs and downstream apoptotic events. Therefore, if the hypothesis is correct, AQP8 deficiency in GCs will lead to prevention of antrum formation, which is contradictory to the evidence that AQP8 deficiency benefits antrum formation.

In the present study, we provided experimental evidence of the potential mechanisms of follicular antrum formation; specifically, GC proliferation, migration, and water transport may participate in this developmental event. In a growing preantral follicle, proliferation and migration of GCs are major forces of follicle enlargement. However, these functions are decreased in an AQP8^-/-^ preantral follicle, probably resulting from the reduced number of GCs in the follicle compared with that in WT. For unknown reason, fluid fills the AQP8^-/-^ preantral follicle so that it is not smaller than the WT follicle in size. The most important changed characteristic of the AQP8^-/-^ preantral follicle was the increased intercellular space or looseness of GCs (**Figure 6**). This characteristic perhaps promotes the occurrence of small foci or gaps in GC mass, which is the beginning of formation of the antral cavity. This hypothesis could partly explain why AQP8^-/-^ antral follicles more easily form antra than WT follicles.

Once the antrum forms in a follicle, the growing follicular size depends on the size of the antrum. In this study, the diameter of AQP8^-/-^ antral follicles was nearly the same as that of WT in ovary sections (**Figure 1C**) and the *in vitro* 3D culture system (**Figure 2D**), suggesting a similar rate of antral enlargement. There are disputes regarding how liquid enters the antrum. McConnell et al. (2002) described that AQP7–9 expressed in rat GCs mediate water movement from outside of the follicle into the antrum via the intracellular pathway. On the contrary, two other earlier studies have suggested that the intercellular space of GCs may be the actual path of liquid flow (Gosden et al., 1988; Clarke et al., 2006). The observations in the present study seem to agree with the latter, but further studies are needed to confirm this.

## Conclusion

We identified increased formation of antral follicles in AQP8^-/-^ mice and *in vitro* 3D-cultured AQP8^-/-^ follicles. The AQP8^-/-^ GCs were impaired in the functions of proliferation and migration, but not steroidogenesis. Such alteration of GC functions led to an increase of intercellular space in AQP8^-/-^ preantral follicles, which is advantageous in antrum formation. The present study elucidated the progression of the essential roles of AQP8 in folliculogenesis and provides novel evidence and insight into the mechanisms of follicular antrum formation.

## Author Contributions

WS designed theexperiments and analyzed the experimental results. DW carried out the experiments and wrote the manuscript. BS, CJ, DZ, GZ, HZ, JH, JW, MS, ML, TM, XD, XM, and YH helped to finish the experiments and the manuscript.

## Conflict of Interest Statement

The authors declare that the research was conducted in the absence of any commercial or financial relationships that could be construed as a potential conflict of interest.
